# Practical aspects of NGS-based pathways analysis for personalized cancer science and medicine

**DOI:** 10.18632/oncotarget.9370

**Published:** 2016-05-14

**Authors:** Ekaterina A. Kotelnikova, Mikhail Pyatnitskiy, Anna Paleeva, Olga Kremenetskaya, Dmitriy Vinogradov

**Affiliations:** ^1^ Personal Biomedicine, Moscow, Russia; ^2^ A. A. Kharkevich Institute for Information Transmission Problems, Russian Academy of Sciences, Moscow, Russia; ^3^ Institute Biomedical Research August Pi Sunyer (IDIBAPS), Hospital Clinic of Barcelona, Barcelona, Spain; ^4^ Lomonosov Moscow State University, Leninskie Gory, Moscow, Russia; ^5^ Center for Theoretical Problems of Physicochemical Pharmacology RAS, Moscow, Russia; ^6^ Orekhovich Institute of Biomedical Chemistry, Pogodinskaya, Moscow, Russia; ^7^ Pirogov Russian National Research Medical University, Ostrovityanova, Moscow, Russia

**Keywords:** next generation sequencing (NGS), systems biology, precision oncology, personalized medicine, pathways

## Abstract

Nowadays, the personalized approach to health care and cancer care in particular is becoming more and more popular and is taking an important place in the translational medicine paradigm. In some cases, detection of the patient-specific individual mutations that point to a targeted therapy has already become a routine practice for clinical oncologists. Wider panels of genetic markers are also on the market which cover a greater number of possible oncogenes including those with lower reliability of resulting medical conclusions. In light of the large availability of high-throughput technologies, it is very tempting to use complete patient-specific New Generation Sequencing (NGS) or other “omics” data for cancer treatment guidance. However, there are still no gold standard methods and protocols to evaluate them. Here we will discuss the clinical utility of each of the data types and describe a systems biology approach adapted for single patient measurements. We will try to summarize the current state of the field focusing on the clinically relevant case-studies and practical aspects of data processing.

## INTRODUCTION

The concept of molecular mechanisms, affected differently in different cancer patients, has been considered as a key to the correct personalized cancer treatment choice [1, 2]. The common way to assess these differences is to take into account markers of certain pathway activity and therapy response. Around 50 of tumor-specific predictive labels have been approved for the companion diagnostics of cancer treatment [3, 4]. In some cases, strong hereditary genetic markers (i.e germline mutations in BRCA1/2) could lead to preventive surgical tissue resection, resulting in a reduced risk of tumor appearance [5, 6].

In addition to the relatively simple single-gene tests widely used by practical oncologists, targeted sequencing panels that include several tens or hundreds of cancer-related genes are also available on the market. They could be used for clinical decisions and are offered by companies like Caris, Foundation Medicine, Personal Genome Diagnostics and others [7, 8] (Table 1). These extended tests mainly rely on the NGS technologies [9, 10] with high coverage of genomic regions of interest and can better describe molecular changes potentially leading to disturbance of cancer pathways.

**Table 1:** Companies, technologies and kits for precision oncology

Name	Website	Disease/Tissue specificity	Scope of coverage and methods	Description (detection with or without therapeutic interpretation)
Foundation Medicine	www.foundationmedicine.com/	Universal cancer panels: Solid tumors (Foundation one) and hematologic tumors (FoundationOne Heme)	Panels	Analysis of solid and Hematologic tumors–detection and interpretation of all class of genomic alterations (including base substitutions, InDels, CNAs, rearrangements and fusion genes)
Personal Genome Diagnostics (PGDX)	www.personalgenome.com/	Universal cancer Panels and tissue specific panel for NSCLC (LungSelect)	Full exome + panel (120 cancer genes)	Detection and interpretation of SNVs, InDels, CNAs and rearrangements
Ambry Genetics	www.ambrygen.com/	Universal cancer panels (solid tumors) + tissue specific cancer panels (ColoNext; OvaNext; PancNext; PGLNext; RenalNext)	Kits and cancer panels + exome and (mtDNA) genome	Detection and interpretation of gene SNVs, InDels, CNAs large rearrangements for specific types of cancer.
GeneDx	www.genedx.com	Universal cancer panels + tissue specific cancer panels (for breast, ovarian, colorectal, pancreatic, endometria cancers and Familial Cutaneous Malignant Melanoma)	Full exome (WES, NGS) + panels	Detection of SNVs, InDels. Deletion testing of mtDNA, detection of mtNDA SNVs.
NeoGenomics Laboratories	www.neogenomics.com/	Tissue specific cancer panels (for NSCLC; Melanoma; Colorectal Cancer)	Panels + IHC, FISH, Flow Cytometry, RT-PCR	Detection of SNVs, InDels, CNAs, rearrangements, fusions
Caris	http://www.carislifesciences.eu/solid-tumours	Universal cancer panel	Panels + IHC; CISH; FISH; RT-PCR; Sanger Sequencing, Pyro Sequencing; Fragment Analysis	Detection and interpretation of SNVs, CNAs, InDels, fusions and level of expression of protein biomarkers in solid tumors for therapeutic decision support and clinical trials matching.
Myriad Genetics	https://www.myriad.com/	Tissue specific cancer panels (breast and ovarian cancer)	Panel for BRCA1, BRCA2	Detection of gene mutations
Quest Diagnostics	www.questdiagnostics.com/	Universal cancer panel	Panel	Detection of SNVs and InDels;
GPS@WUSTL	http://gps.wustl.edu/	Universal cancer panels	Panel	Detection of SNVs and InDels
Arup Laboratories	https://www.aruplab.com	Tissue specific cancer panels for gastrointestinal cancer	Panel + IHC, FISH, and PCR	Screening, risk prediction, diagnosis, prognosis, monitoring, pharmacogenomics, and therapeutic triage of malignancies. Detection of SNVs, InDels, chromosomal alterations and level of expression of oncomarkers.
MolecularHealth	www.molecularhealth.com/	Universal cancer panels	Exome+panel (over 500 cancer-related genes), Comparative Genomic Hybridization (aCGH) used as an additional test	Interpretation of whole exome analysis data, detection and interpretation of gene alterations. Integration and interpretation of biological, medical and drug Response information.
Personalis	www.personalis.com/	Universal cancer panels (solid tumors)	Panels (more than 1,300 cancer genes and more than 200 miRNA genes)+ exome (WES)+ transcriptome	Detection of SNVs, InDels, CNAs, fusion genes, LOH, gene expression profiling, low-level variant expression.
OncoDNA	www.oncodna.com/	Universal cancer panels (solid tumors)	Panels (OncoDEEP DX - 65 genes, with wide coverage of the KRAS, BRAF, EGFR; OncoDEEP Clinical - more than 400 genes; Plus Package - multi-platform approach to complete the characterization of the tumor, including FISH, PCR, ICH)	Detection and interpretation of SNVs, InDels, CNAs, translocations, microsatellite instability, DNA methylation, presence and activation of specific proteins. Integration of all the data, analysis of molecular networks, findings of the latest publications and generation of a comprehensive and intuitive report.
GenomOncology	www.genomoncology.com/	Universal cancer panels (solid tumors)	Bioinformatic service	Interpretation of NGS data (SNVs, InDels, CNAs, translocations and other structural variants) and translate the specific molecular profile of each patient's tumor genome into an actionable clinical report.
MI-ONCOSEQ Study (Michigan Oncology Sequencing Center, University of Michigan)	http://mctp.med.umich.edu/physicians/mi-oncoseq-study	Universal cancer panels (solid tumors)	WES + transcriptome sequencing	Detection and interpretation of tumor somatic and germline SNVs, InDels, CNAs, gene fusions and rearrangements, gene expression alterations.
Genewiz	www.genewiz.com/	Cancer (solid tumors)	Cancer panels (OncoGxOne™+ Hot spot cancer panels), exome sequencing, whole genome sequencing, transcriptome (RNA-Seq)	Detection and interpretation of SNVs, InDels, CNAs, rearrangements, low-frequency aberrations, gene fusions, transcriptome analysis, identification of splice variants.
Neogenomics Laboratories	www.neogenomics.com/	Universal cancer panels (solid tumors)	Panels	Detection and interpretation of genomic alterations including SNVs, InDels, CNAs.
Emory Genetics Laboratory	http://geneticslab.emory.edu/index.html	Universal cancer panels (solid tumors)	WES + Panels	Detection and interpretation of Exome data: SNVs, InDels.
Paradigm Cancer Diagnostic (PCDx)	http://www.paradigmdx.org/	Universal cancer panels (solid tumors)	Exome, transcriptome (over 500 cancer-related genes)	Detection and interpretation of a patient tumor SNVs, CNAs, InDels, rearrangements and fusions, mRNA expression and protein expression.
Rosetta Genomics	www.rosettagenomics.com/	Universal cancer panels (solid tumors)	Transcriptome	Detection only. microRNA-based diagnostics service.
ThermoFisher	https://www.thermofisher.com/	Universal cancer panels (solid tumors)	Exome	Detection of SNVs, InDels, CNAs and gene fusions.
Swift Bioscience	www.swiftbiosci.com/	Universal cancer panels (solid tumors)	Kits for Illumina NGS and Ion Torrent Platforms; TP53 panel for Illumina Platform	Detection of genes aberrations: SNVs and methylation status, from variety of clinical sample types.
Illumina	http://www.illumina.com/content/dam/illumina-marketing/documents/products/other/cancer-panel-comparison-tool.pdf	Universal cancer panels (solid tumors)	Panels	Detection of germline or somatic SNVs in solid and myeloid tumors.
Asuragene	http://asuragen.com/	Universal cancer panel	Pan cancer kit QUANTIDEX™	Detection of the scope of variants reported by the panel including >1,600 known COSMIC variants, SNVs, InDels, and structural rearrangements targeted by the panel.
RainDance Technologies	http://raindancetech.com/	Universal cancer panel + Tissue specific panels (for acute myeloid leukemia (AML), myelodysplastic syndromes (MDS), myeloproliferative Neoplasms (MPN), myeloma.	Panels: ThunderBolts™ Cancer Panel (Interrogate mutations/hotspots in 50 oncogenes, tumor suppressors and drug resistance markers); ThunderBolts™Myeloid Panel (Target mutations/hotspots in 49 genes implicated in AML, MDS, MPN and myeloma diseases, including challenging genes such as CEBPA and NOTCH1.	Detection of SNVs in cancer related genes.

Increasing availability of genetic testing facilities has led to the so-called basket trial studies, where selection of patient-specific cancer treatment is defined by tumor molecular profile, but not by its tissue of origin [11–14]. However, some researchers suggest that one should not consider only tumor gene alterations but also take intо account its tissue specific features. [15–17] (Table 1).

The most comprehensive NGS-based cancer studies would implicate the screening of germline genome [11], tumor genome [11, 12], transcriptome [13] and methylome [14] in the search of potential cancer driving alterations [15]. Genome screening can be performed by capturing cancer-related genes only (NGS-based cancer panels), all transcribed regions (whole-exome sequencing, WES) or whole-genome sequencing (WGS) [16]. Despite the tempting idea of WGS usage and the decreasing cost of NGS technologies, large-scale WGS studies are still unaffordable for many research laboratories and clinical settings. Thus, targeted cancer panels with high coverage of the selected genes or WES with restricted coverage of all genes, depending on the tasks, could be the preferable choice of DNA analysis.

In 2013, the U.S. Food and Drug Administration (FDA) approved the use of the Illumina MiSeqDx platform for high throughput NGS in clinics [17]. This decision by the FDA has paved the way for future clinical diagnostic and prognostic use of NGS and the emergence of the Precision Oncology 3.0 paradigm [18]. Precision Oncology 3.0 encourages the usage of systems biology, including pan-omics data and reverse engineering methods for hypothesizing the putative molecular networks that drive a given patient's tumor and for the selection of cancer or non-cancer off-label therapies that are potentially beneficial in the studied case. This approach, despite the presence of dosage, toxicity, efficacy and ethical issues, could be a promising strategy for oncologists to choose between the available therapies or to provide an alternative treatment regimen to the patients unresponsive to the standard care in order to improve therapeutic response and to minimize adverse events [19].

At the moment there are more than 50 web-sites that suggest different approaches to personalized cancer care [20]. Most of these cancer care organizations are using NGS-based targeted sequencing panels (Table 1) for studies of cancer driving SNVs (Single Nucleotide Variants) and InDels (Insertions and Deletions), while Fluorescence in situ hybridization (FISH) [21–23] and immunohistochemistry [24](IHC) are the standard methods of choice for the detection of cancer-related translocations and specific expression markers. Despite the fact that it is also possible to identify all the above-mentioned clinically-relevant molecular events using NGS methods (Table 2), the gold standards of NGS data processing for cancer samples are still under development [25]. There remain a lot of problems to be solved, the main concept to be proved and the confirmed designs of the Precision Oncology 3.0 clinical trials to be defined.

**Table 2:** Main clinically relevant cancer events, detectable by NGS

Event type	Sample type	Tissue type	References
Germline mutations (SNV/InDel)	DNA, RNA	Control tissue or blood	[192–195]
Somatic mutations (SNV/InDel)	DNA, RNA	Tumor and control tissue (or blood)	[61,196–199]
Somatic copy number alterations (CNA)	DNA	Tumor and control tissue (or blood)	[200–202]
Gene fusions and other somatic structural variations (SV)	DNA, RNA	Tumor and control tissue (or blood)	[23,203,204]
Methylation pattern changes	DNA	Tumor and control tissue	[14,77,205]
Differential gene expression	RNA	Tumor and control tissue	[73,206]
Differential alternative splicing	RNA	Tumor and control tissue	[73,207]

## ENCOURAGING CASE STUDIES AND DESIGN OF CLINICAL TRIALS

The increasing attention to the field of precision oncology is supported by encouraging NGS-based personalized treatment guidance case studies. Among the first was a case of 78-year-old male patient, diagnosed with adenocarcinoma of the tongue published in 2010 by Jones et al. [26]. The patient went through erlotinib treatment without a positive effect and had to go through further therapy. Analysis of omics data (genome and transcriptome sequencing from tumor and normal tissues), using Ingenuity Pathway Analysis software [27], KEGG pathways [28] and the DrugBank [29] drug target database, revealed gene expression changes relevant to the signaling pathways involved in cancerogenesis. Two potential driver genes, up-regulated RET and down-regulated PTEN, that were probably related to the ineffectiveness of erlotinib were found. Once the therapy was changed to sunitinib, the volume of the tumor started to decrease. However, after 5 months the tumor started progressing again and the patient was transferred to sorafenib and sulindac as alternative drugs. Next, genome and transcriptome sequencing of tumor samples from metastasis was performed. Omics data analysis revealed nine *de novo* mutations not present in the controls nor in the tumor samples prior to the therapy. Further exploration suggested that resistance to sunitinib and sorafenib could be explained by the acquired upregulation of both MAPK/ERK and PI3K/AKT pathways. Eventually, this analysis of omics data led to the hypothesis that only a cocktail of targeted drugs would be able to reduce the proliferation of the tumor cells. The authors additionally speculated that as sequencing costs continue to decline, whole genome characterization will become a routine part of cancer pathology.

Welch JS et al. [30] in 2011 has described the use of WGS in “real-time” diagnosis and detection of an oncogenic fusion gene created by an insertional event. Within seven weeks, the authors had completed the process of library generation, massive parallel sequencing, analysis, and validation of a novel fusion that created a classic PML-RARA bcr3 variant. These findings altered the medical management of the patient, who then received all-trans retinoic acid instead of an allogeneic stem cell transplant.

One of the most inspiring examples was published not in a scientific article, but in the “New York Times” journal [31] in 2012. Oncologist himself, Dr. Lukas Wartman was diagnosed with the same type of tumor that he studied, Acute Lymphoblastic Leukemia (ALL). He was treated with chemotherapy and received necessary stem cell transplants. That put him back in remission, but in several years he relapsed again with only 4 or 5 percent chance of survival. Whole genome and whole transcriptome sequencing was then performed at the same institution where Dr. Wartman worked. The actionable modification (overexpression of FLT3) was found, and the drug (sunitinib or Sutent) approved for treating advanced kidney cancer, was administrated at his own risk. After the treatment, the patient went into full remission and, according to the Washington University School of Medicine in St.Louis web-page, returned to his work as an Assistant Professor in Oncology, at the time this review was written.

Other successful clinical examples of genetic analysis for personalized medicine were published in 2014 by Caris company[32]. Using the Caris Molecular Intelligence (CMI) platform - a combination of genome sequencing, FISH (fluorescence in situ hybridization method) and PCR the authors analyzed two patients. The first was a 63-year-old man with the progressive metastatic prostate cancer, which caused considerable pain. The researchers identified decreased expression of thymidylate synthase (TS) in the tumor. Since low TS expression is known to be associated with tumor sensitivity to fluoropyrimidines and other folate analogs, the drug therapy - pemetrexed - had been prescribed. As a result, the size of the metastases was reduced and the tumor PSA marker, LDH, was normalized. The patient's condition returned to normal. The treatment has been tolerated exceptionally well and no further admissions to the hospital became necessary. The second patient was a 49-year-old woman diagnosed with stage IV ovarian cancer. Surgery confirmed metastatic disease and the patient began standard treatment with a combination of intravenous paclitaxel and carboplatin and intraperitoneal docetaxel/cisplatin. During the time of that treatment, the patient had a partial response. Biopsy material was sent for CMI testing to identify any additional treatment options. The CMI report indicated potential benefit from the combination treatment of irinotecan and cetuximab based on the expression profile of the patient's tumor. This combination decreased the level of the patient's cancer antigen 125 to normal and allowed it to stay normal over the course of the first 8 months of treatment. Unfortunately, toxicity effects led to the discontinuation of the therapy. However, the demonstration of the efficacy irinotecan and cetuximab, which are rarely used in ovarian cancer treatment, is of significant importance as it justifies further exploration of treatments guided by tumor profiling instead of using the histological diagnosis of the tumor alone.

Among the most important up-to-date advances in current Precision Oncology, one could name the massive molecular-profiling-based clinical trial studies published by researchers from France [33, 34] and the USA [35–37] and multinational consortium WINTHER [38].

One of these studies, a whole-exome sequencing precision medicine trial that captures a diverse range of patients with advanced treatment-resistant cancer and prospective 7-25 months clinical follow-up, was published by Beltran H et al. [36]. More than 90% of the patients were shown to harbor actionable or biologically informative alterations, although treatment guided by this information was only present in 5% of the cases because of the lack of patient access to clinical trials and/or off-label use of drugs. Similarly, the feasibility study published by the French researchers [34] is the first proof-of-concept multicentric randomized clinical trial (SHIVA) comparing targeted therapy based on tumour molecular profiling *vs* conventional therapy in patients with refractory cancer. The druggable molecular abnormalities on the level of mutations, gene copy number alterations or IHC analyses were found for the 38 out of 100 enrolled patients with metastatic cancer who failed standard therapy.

Other modern approaches to clinical trials, extensively sponsored by National Institute of Cancer (NCI), include both “genotype to phenotype” and “phenotype to genotype” initiatives [39]. In particular, the molecular profiling-based assignment of cancer therapy is a goal of clinical trials NCI-MATCH [40] and NCI-MPACT [37, 41]. One of the examples of “phenotype to genotype” initiatives is “Exceptional Responders”, a clinical trial inspired by previous case reports [42, 43]. It implies a retrospective analysis of tumor molecular features that may explain why patients responded particularly well to a particular treatment.

Multinational clinical trial WINTHER (five countries, six sites, coordinated by the Worldwide Innovative Networking Consortium) used genomic assays in making treatment decisions. This trial has been launched in order to assess the efficacy of therapy determined by matching of “genomic diagnosis” with targeted drugs [38].

These trials are the first ones to use a randomized design to examine whether assigning treatment based on genomic tumor screening can improve the rate and duration of response in patients with advanced solid tumors. Despite the fact that efficacy results for them are not available yet, the organization of the corresponding pipelines and clinical trial settings is extremely important for further advances and clinical validation of precision medicine approaches [15].

## SYSTEMS BIOLOGY PIPELINE FOR THE CANCER-RELATED NGS DATA

Systems biology is a holistic rather than a reductionist approach to understanding and controlling biological complexity [44, 45]. Using systems biology, researchers obtain, integrate and analyze complex datasets from multiple experimental sources and molecular levels using interdisciplinary approaches. Being applied to the cancer research, the goal of systems biology is to decipher the impact of genetic and epigenetic aberrations in cancer cells onto their homeostasis, intercommunication and response to the possible treatments [46].

In systems biology, the scientific community is mainly focused on statistical approaches [47] and on trying to identify the features characteristic for the specific group of patients, and not on the concept of studying individual patient. As a consequence, some of the available tools in the field are not appliable for truly personalized studies. For example, pathways identified by gene expression profiling using group analyses differ considerably in comparison to those identified by personalized analyses [48]. However, the systems biology approach is specifically important for precision oncology [49], since each tumor is unique in terms of genetics, epigenetics and pathological rewiring of signaling pathways. Modeling of patient-specific molecular processes could help medical doctors identify the most effective treatment, minimize toxicity and avoid unnecessary trials and errors.

To start the NGS-based systems biology pipeline, it is necessary to obtain DNA and/or RNA samples from the studied tissues. The most widespread type of samples is the fresh-frozen paraffin embedded (FFPE) block, although blood sample or fresh surgical material could also be used upon availability and tasks. Thus, DNA or RNA, extracted from the blood [50, 51] as well as from FFPE [52, 53] or fresh tissue [54, 55] could be used for the detection of genetic alterations. Since transcriptomics studies are more prone to tissue-specificity, RNA from FFPE samples or fresh tissues of the same origin is necessary for reliable identification of gene expression changes.

**Figure 1:**
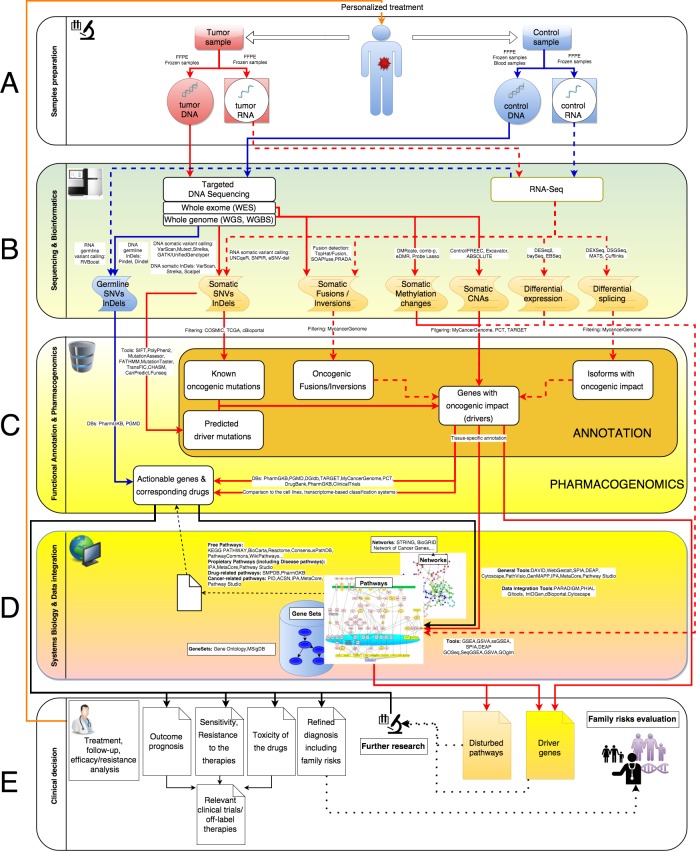
Generalized systems biology pipeline for the cancer-related NGS data processing Here, solid blue and red lines correspond to DNA processing while dashed ones to RNA processing. Blue lines represent germline events, red ones - somatic. **A.** Sample preparation. Extraction of DNA and RNA from patient's tumor and normal tissue. **B.** Sequencing and Bioinformatics. Convert raw sequencing data into list of genetic variations. **C.** Functional annotation and Pharmacogenomics. **D.** Systems Biology and data integration. **E.** Clinical decision

Just after the sample preparation and detection by the best applicable sequencing techniques, many steps of data analysis have to be performed to obtain personalized clinically-relevant information. In general, these steps can be classified into 3 main categories: Sequencing & Bioinformatics, Functional Annotation & Pharmacogenomics and Systems Biology & Data integration. Generalized systems biology pipeline for the cancer-related NGS data processing is represented on Figure 1. Here we assume that all tasks necessary to identify potentially clinically relevant events (like SNVs calling, CNAs detection and so on, see Table 1), starting from read counts, could be addressed as bioinformatics tasks. The relevance of these events to cancer progression and response to the therapies could be inferred either by direct application of already available event-specific tools and databases (Functional Annotation & Pharmacogenomics), or by more sophisticated Systems Biology& Data integration approaches. Below we describe these categories in details.

## SEQUENCING AND BIOINFORMATICS

Nowadays NGS is one of the most common high-throughput technologies due to its relatively low cost and high efficiency in processing of genetic material. The detailed description of NGS products and technologies is out of the scope of this paper but is thoroughly reviewed elsewhere [56, 57]. As with all high-throughput experimental methods, the data processing step is critical for obtaining correct results. Moreover, the impact of this step in the total cost of a sequencing project is steadily rising, while the share of experimental expenses is falling significantly [58]. Here we will cover main aspects of the NGS data processing, relevant to precision oncology.

The goal of the bioinformatic analysis can be expressed as ‘to provide the exhaustive list of genetic features that are related to the occurrence of cancerous phenotype in the given sample’. This general task can be divided into several specific steps that are 1) NGS reads trimming and quality control; 2) mapping to a pre-built assembly; 3) alignment cleanup; 4) variant detection (or, broadly speaking, genome annotation). While the first three steps are rather technical (though important) and usually performed with small deviations from the generally common way, the last one is specific for each variation type. In Table 3 we summarize the information that can be useful during this stage (i.e. lists of the popular tools, noteworthy difficulties and some of the clinical implications), while a more detailed explanation is provided further.

**Table 3:** Tools used for different modification types prediction

Variation type	Single sample variant detection tools	Somatic variant detection tools	Difficulties	Clinical usefulness
SNV DNA	VarScan[193], GATK [66]	VarScan [193], Mutect [208], Strelka [209], GATK: UnifiedGenotyper [66]	High coverage is required for mutations with low allelic fraction. Reference bias.	Used by most of the approved genetically-based drug indications
SNV RNA	RVBoost [197]	UNCqeR [62], SNPiR [198], eSNV-detect [210]	Without DNA data can be confused with RNA-editing sites. Insufficient coverage for weakly expressed genes.	Provides extra layer of information whether mutated gene is expressed
InDel	Pindel [211]^1^, Dindel [212]	VarScan [193], Strelka [209], Scalpel [213]^2^	Surrounding SNVs can prohibit correct read alignment.	Can greatly impact protein function by inducing a frameshift or deleting a domain
CNA	EWT [214], CNV-seq [215], FREEC [216]	Control-FreeC [200], BICseq [217], Excavator [201], ABSOLUTE [218]	Low boundary precision when used on WES data [67]	Help in driver genes detection [219], can be linked with outcome prognosis [220]
Fusions	TopHat-Fusion [221], SOAPfuse [222]	SOAPfuse [222], PRADA [223]	Validation is highly recommended. Can be confused with splicing aberrations.	Often linked very tightly with a specific disease, thus alleviating diagnosis
Differential methylation	N/A	DMRcate [224], comb-p [225], eDMR [226]	Experimental costs are rather high. Low coverage of all genomic CpG sites for some methods.	Can be used as biomarker for prognosis and therapy response prediction [227]
Differential expression	N/A	DESeq2 [74], Cufflinks [73], baySeq [75], limma [228]	Reliable prediction requires several replicates for both tissues. Сontrol sample should be of the same origin as the tumor	May be used for diagnosis, prognosis, therapy response prediction and monitoring [229,230]
Differential splicing	N/A	DEXSeq [231], DSGSeq [232], MATS [233], Cufflinks [73], limma [228]	Requirements for replicates count are higher than for expression analysis. Rare splicing events detection needs high coverage.	May provide information for development of diagnostic tests, evaluating therapy efficacy [234], with potential application as prognostic and predictive markers [235]

1 There are modifications by third-party that allows search for somatic indels

2 Method used for searching somatic InDels is not mentioned in original paper

N/A - not applied.

The most frequent type of modifications is SNV (single nucleotide variations) [59]. Though many software packages have been developed for somatic SNV detection, the problem is far from being solved. The main source of difficulties is the low allelic fraction of mutations caused by tumor heterogeneity and polyclonality. Combined with practically reachable read coverage level this makes a large fraction of mutations indistinguishable from sequencing errors. Several recent papers note the high level of inconsistency among different tools predictions [60, 61]. To solve this issue, some authors propose simultaneous usage of several programs, which was proved to be advantageous [60]. Another problem is the phenomenon called ‘reference bias’- disposition of popular read mapping tools to discard or place incorrect reads with alternative alleles. Since reads with reference alleles are not affected, this leads to a decrease in maximum possible sensitivity, especially in weakly covered regions. One possible solution for this problem is to perform sequencing of RNA instead of DNA. The obvious benefits are higher coverage level for modestly expressed genes and potentially higher impact of all SNVs (mutations in non-expressed genes are less likely to be drivers). The main obstacles are the phenomenon of RNA-editing, which can lead to the appearance of false-positive calls in results, and low or zero coverage for weakly expressed alleles and regulatory regions. The best choice seems to be simultaneous sequencing of both DNA and RNA [62].

The second most common type of clinically useful events are short insertions and deletions (often referred as InDels). Modern read mappers often provide incorrect alignment in regions surrounding InDels, leading to a noticeable rise of error rate for both SNV and InDel calling [63, 64]. Thus realignment of reads in these regions is a crucial step of the bioinformatics analysis. Nevertheless it is not performed automatically by most popular read mappers because of its computational complexity. Some tools (e.g. ABRA [65] and HaplotypeCaller from GATK package [66]) incorporate another strategy - instead of mapping reads to reference genome, they perform local de-novo assembly.

While a single SNV or InDel act only on one gene, a CNA or a SV usually affects several of them. The process of detecting a set of CNA can be divided into three stages: estimating the copy-number for each locus, detecting true CNA boundaries by merging neighboring loci and then classifying the resulting CNAs. The first stage requires precise information about the local sequence properties in order to correct possible biases of the sequencing technology and the read mapping tool. Inferring proper boundaries is greatly impaired when WES strategy is used compared to WGS with accuracy being reduced up to two orders of magnitude [67]. For review of computational methods applicable to CNAs detection, see [68].

Gene fusions can often be heavily correlated with a specific cancer subtype (i.e. pathognomonic) or choice of targeted therapy. For example in a recent study [69] all patients with fibrolamellar hepatocellular carcinoma were found to have DNAJB1 and PRKACA genes fused, while no patients with other kinds of liver neoplasia had this modification. Additionally, gene fusions BCR-ABL and EML4-ALK are predictive markers to imatinib [70] and crizotinib [71] treatments, respectively. Therefore, fusion detection should be considered a crucial part of the diagnostic procedure. As SNVs and indels, gene fusions can be explored using either DNA or RNA data. RNA sequencing has several deficiencies (low expression levels of some fusions, inability to detect variations in regulatory regions), so the optimal strategy, again, seems to be the simultaneous usage of DNA and RNA data [72].

Detection of differentially expressed genes between two tissue samples can be considered a quite mature area itself. Many methods were developed even before the advent of NGS technology in order to process expression data from hybridization microarrays, and while RNAseq expression data differs significantly in some aspects, the general idea mainly stays the same. Several of the most popular software packages include Cufflinks [73], DeSeq 2 [74] and baySeq [75]. And yet, some problems still persist. One of them is the very wide dynamic range of gene expression levels, which can yield noticeable bias in the results. Another source of issues is the presence of alternative splicing. Different transcripts of the same gene often perform quite distinct functions, which makes it important to separate gene isoforms during expression analysis. In its turn, detection of differences in alternative splicing events is complicated by the incomplete description of the splicing process even in healthy tissues.

Compared to expression analysis, evaluation of methylation data is rarely performed in cancer studies. Whereas the functional significance of DNA methylation for cancer has long been proved [76], genome-wide studies using comprehensive methods are still quite scarce [77], probably due to the high cost of experiments. In one of the recent examples, Stirzaker et al. suggests a possible connection of methylation patterns with outcome prognosis for triple-negative breast cancer [14].

Nowadays, next-generation sequencing (NGS) can also be used as a powerful tool for identification of rare events, e.g mosaicism. Some mutations acquired early in embryonic development that may be involved in cancer predisposition can be missed by less sensitive technologies [78]. The crucial point is the ability to detect low levels of mosaicism while accounting for the importance of tissue-specific mosaicism in disease and the potential increase of mosaicism frequency rate with age [79,80]. Mosaicism detection is important for individuals in the risk group or diagnosed with cancer. NGS based genetic testing may demonstrate levels of mosaicism much higher than the previously expectedfrequency. Mosaicsm may be observed in certain cases evenwithout apparent familial cancer history, as was demonstrated for gene APC and FAP (Familial adenomatous polyposis) patient, and BRCA in breast cancer patient. Today this approach is not widely applied in routine clinical practice and reports of somatic mosaicism detection are limited [81–83].

## FUNCTIONAL ANNOTATION AND PHARMACOGENOMICS

A typical cancer sample contains several dozens of somatic mutations that may alter the functioning of the corresponding proteins. However, a relatively small fraction of genetic alterations leads to a small selective advantage of cancer cells and hence stimulates the tumor growth. Such alterations are called driver mutations [84], and their number is usually somewhere between two and eight per a tumor sample [19]. A subset of mutations may be “actionable”, i.e. may have significant diagnostic, prognostic, or therapeutic implications in subsets of cancer patients [85]. On the contrary, the majority of the somatic mutations, so-called passenger mutations are a byproduct of the unstable cancer genome, and tend to not affect the fitness of tumor cells. Thus, they cannot serve as diagnostic and prognostic biomarkers [86]. However, there is some evidence that passenger mutations can be deleterious to cancer cells, altering the course of a tumor progression [87].

Since driver mutations provide growth advantage for the cancer cells, the most intuitive strategy to identify driver genes is to detect signals of evolutionary positive selection across tumor samples. Various approaches to quantify different evidences of selection pressure have been proposed. For example Tamborero et al. [88] employed several complementary methods including searching for genes with significant differences in mutation rates or enriched with mutations showing high functional impact, significant regional clustering or affecting phosphorylation-associated sites. This large-scale meta-analysis performed across 3,205 tumors produced list of 291 high-confidence driver genes. A similar type of analysis across 21 tumor types was done by Lawrence et al. [89] and integrated three independent signal types including enrichment of mutations in evolutionarily conserved sites. A total of 254 genes were identified.

Positively selected driver mutations are more likely to recur across multiple patients and tumor types [16]. Hence the first level of the event annotation may consist of filtering found somatic alterations and the identification of the previously reported ones. Recent advances in NGS methods have led to accumulation of thousands of publicly available cancer genomes. There are several sources of the relevant information, including the Catalogue of Somatic Mutations in Cancer, COSMIC [90] and the Cancer Genome Atlas, TCGA [91]. These huge amounts of data can be easily summarized with the help of other resources, such as cBioportal [92] or UCSC Cancer Genomics Browser [93]. However, frequency-based analysis has certain limitations in detecting driver mutations. Although several well-established cancer genes are mutated in a high proportion of tumours (like TP53, KRAS, BRAF, PTEN), most genes are mutated at intermediate and low frequencies (2-20%) [89].

It is important to emphasize the difference between predicting driver genes and individual driver mutations. Not all the alterations in the cancer-associated driver gene can be treated as driver mutations. An alteration in a proto-oncogene can be considered a driver mutation only if it leads to gene activation or results in a new function. Similarly, to claim a mutation is a driver, it should clearly impair functioning as a tumor suppressor. Hence, many driver mutations have too low occurrence levels to be detected only by frequency-based analyses using currently available data. It will therefore be necessary to employ algorithms for driver/passenger prediction which consider the mutations’ local functional and genomic context.

The challenge is to differentiate between driver and passenger mutations and rank the former according to their likelihood of promoting tumor progression. It is important to distinguish between individual alteration driver/passenger discrimination and prediction of the mutation impact upon a protein function. Many computational tools have been developed for the latter problem including SIFT [94], PolyPhen2 [95], MutationAssesor [96], FATHMM [97] and MutationTaster [98]. Although they were not intended for predicting driver mutations, these algorithms can be used to filter out variants that are unlikely to affect the structure and function of the protein, i.e. have more chance to be passenger mutations. For in-depth reviews of remaining challenges in the field of driver identification including prioritization of variants within the non-coding regions please refer to [99–100].

Some tools including FATHMM and MutationAssessor claim to assign higher functional impact scores to mutations occurring in driver genes. The former algorithm also has a special version in which a cancer-specific weighting scheme was incorporated to potentiate the functional analysis of driver mutations [101]. Similar approach is adopted in TransFIC software [84] where the scores obtained *via* PolyPhen2, SIFT and MutationAssessor are transformed in order to discriminate likely drivers from likely passengers. For each somatic mutation, its prediction score is compared with the distribution of scores for germline mutations located within functionally-related genes. Observed significant differences suggest that the mutation under study may be involved in cancer development.

Several algorithms like CHASM [102] and CanPredict [103] treat the differentiation between driver and passenger somatic missense mutations as a classification problem. The random forest classifier is trained to distinguish between driver mutations curated from COSMIC and passenger mutations generated according to background substitution frequencies. Each mutation is described by various features such as amino acid substitution properties, alignment-based estimates of conservation, predicted local structure, and etc.

In Funseq paper [104], instead of binary classification, variants are prioritized according to several criteria including occurrence of mutation in 1000 Genomes Project, breakage of transcription-factor binding site, location within gene under strong selection or in a hub gene and etc. Variants on the top of the list are more likely to be cancer drivers. An important feature of Funseq is its ability to prioritize mutations located in non-coding regions.

Still there is significant room for improvement of tools for driver mutation prediction. Assessment of several algorithms showed that no single method or combination of methods exceeded 81% accuracy [105].

## CLINICAL INTERPRETATION AND PHARMACOGENOMICS

A reliable assessment of driver mutations, though it may help with identifying specific mechanisms of tumorigenesis, still may not have an immediate prognostic or treatment value. Currently, nearly all molecular therapies can directly target only driver genes with activating mutations (typically oncogenes, such as kinases). On the contrary, restoring loss-of-function alterations in many tumour suppressors requires other more complicated strategies like gene therapy [106], inhibition of a functionally connected genе [107], synthetic [108] and collateral [109] lethality. Review of promising approaches aimed at targeting tumor supressors therapeutically can be found in [110].

Results of high-throughput personalized genomic analyses show that conceivably actionable mutations are quite frequent. Jones with co-authors succeeded in identifying and linking somatic alterations in genes with potentially actionable consequences for 77% of cases [111]. This included associations with known therapies and current clinical trials. This estimate is very close to other results, [112] where the percentage of patients predicted to benefit from targeted agents (again, including clinical trials) was 73% of cases. However when considering only FDA-approved therapies (including drug repurposing), this value reduces to 40%, and considering only standard clinical guidelines, this percentage becomes 6%.

In 2014, the FDA included 10 new drugs and biologics in a list containing more than 165 pharmacogenetic labels for approved agents [3,113]. Only around 50 of them are related to oncology and clearly associated with efficacy, but some were for toxicity pharmacogenetic labels [114]. While the number of efficacy pharmacogenetic labels for the new drugs has doubled in the last four years (e.g. crizotinib, ceritinib for ALK rearrangements; bosutinib, omacetaxine and ponatinib for BCR-ABL fusion protein; dabrafenib, trametinib and vemurafenib for mutated BRAF), the number of toxicity markers (e.g. TPMT, DPD, G6PD and UGT1A1 deficiencies) has not grown so fast and generally corresponds to the older therapies [19].

The numbers presented above show a great promise for drug repurposing and illustrate the potential of precision oncology for a large number of patients whose tumors harbor potentially druggable alterations [19,115]. To promote the corresponding studies, several resources with information about clinically actionable somatic mutations were created.

One of the examples is a curated database TARGET (Tumor Alterations Relevant for Genomics-drivEn Therapy) from Broad Institute, storing information for about 135 genes that may have therapeutic, prognostic and diagnostic implications [116]. It includes rationales behind each gene, the types of recurrent alterations that have clinical relevance in these genes, and the potential therapies. The Personalized Cancer Therapy (PCT) resource fromMD Anderson Cancer Center collects associations of genomic alterations with tumor development and growth, changes in response to therapy, availability of FDA-approved drugs, and investigational agents in clinical trials [117]. MyCancerGenome resource [www.mycancergenome.org] matches tumor mutations to targeted therapies including available clinical trials. The paper by Meric-Bernstam et al [117] provides a list of 120 potentially actionable genes for genomically informed therapy, the overlap with TARGET being approximately two thirds.

In addition to tumor-specific alterations which affect therapeutic efficacy, there are a number of germline genetic variants which can result in large interindividual differences in the pharmacokinetic profile of a drug [19]. Genetic alterations in genes responsible for drug metabolism and transport may lead to severe toxicities and should be taken into account by physicians for proper dose adjustment. Common examples of genotype effect and dose on toxicity include polymorphisms in TPMT and thiopurine drugs, UGT1A1 and irinotecan, DPD and 5-fluorouracil. [references]

So far, association between SNVs and drug response is the most studied variant in pharmacogenomics. However, other types of variants including CNAs, InDels and fusions can also guide therapy. A classic example is the use of trastuzumab for HER2-amplified/overexpressing breast cancer [118]. Examples of clinically relevant somatic fusions include EML4-ALK in non-small-cell lung cancer (sensitive to crizotinib [71]) and BCR-ABL fusions in chronic myelogenous leukemia (sensitive to imatinib [70]).

Researchers need to gain a deeper insight into the complex dynamics of subclonal architecture and its impact on disease outcome and prognosis [119]. Genomic stratification of cancers has usually relied on tumor profiling, so it reflects mutations present only in the majority of cancer cells. Intra-tumour clonal heterogeneity can restrict response to therapy, including the emergence of drug-resistant malignant cells and metastasis. Even minor subclones may be clinically relevant. Thus, it has been shown that patients with colorectal cancer harboring KRAS mutations in minor subclones were resistant to anti-EGFR antibodies.[reference]

In addition, resistance may develop in patients who initially responded to therapy. And possibility of evolution of different clones under the selective pressure of therapy has to be taken into account [120].

There are several resources containing information on gene-variant-drug relationships. One of the most authoritative sources is PharmGKB [121], which contains manually curated variant annotations, potentially clinically actionable gene-drug associations, genotype-based dosing guidelines, drug-centered pathways and other pharmacogenetic summaries for most FDA-approved drugs. Genes, for which there are known pharmacogenetic relationships are called VIPs, Very Important Pharmacogenes. Another valuable source of drug-gene interactions that also includes information about anti-neoplastic drugs is Drug-Gene Interaction database (DGIdb) [122]. It integrates data from several sources including PharmGKB, DrugBank, Therapeutic Target Database (TTD) and ClinicalTrials.gov and includes records about known drug targets as well as potentially druggable genes. An example of an NGS-oriented resource is PGMD, PharmacoGenomic Mutation Database from BioBase [123], a manually curated comprehensive collection of all genomic variants that have been reported to have a pharmacogenomic effect in human studies. Online access to PGMD is free for users from academic institutions.

## TISSUE-SPECIFIC ANNOTATION, CANCER CELL LINES, EPIGENETICS

Tumor localization is known to be a strong factor for the observed molecular profile, restricting the application of drug therapy. For example, while BRAF V600-mutated melanomas are sensitive to vemurafenib, BRAF V600-mutated colorectal cancers may not be as sensitive [124, 125]. This problem leads to the necessity to take into account the tissue-specific information. One of the ways to use tissue ‘prior’ is to utilize data on high-throughput characterization of cancer cells, connecting genomic and transcriptomic alterations to drug response pharmacologic profiles. For some cancer localizations different high-throughput-based classification schemes (sometimes non-NGS) have been proposed leading to survival or treatment outcome predictions [126–129]. Ideally it would be perfect to obtain comprehensive omics data across large cohorts of patients but this approach is prohibitively expensive and limited in the scope of drugs that can be tested [130]. Instead, it is much more feasible to perform drug screening coupled with omics-analyses in cell cultures, given that cell line molecular profiles resemble corresponding primary tumours [131].

There are several papers devoted to combining patient data with molecular profiles and drug sensitivity of the cell lines, thereby predicting a possible response to the therapy. Geeleher with colleagues developed ridge regression models for the prediction of chemotherapeutic response in patients based on tumor gene expression and drug IC50 values from a large panel of cell lines [132]. In another study, the authors utilized partial least squares regression-based modeling framework in order to build drug sensitivity models for erlotinib or sorafenib [133]. The cell line panel was used as the training dataset, while the algorithm performance was evaluated using gene expression data from patients treated with the same drug. For a comparative analysis of 44 drug sensitivity prediction algorithms, please refer to [130].

There are several large-scale projects devoted to drug sensitivity of cancer cell lines. Genomics of Drug Sensitivity in Cancer Project [134] contains information about drug sensitivity to 138 anti-cancer therapeutics for more than 1000 human cancer cell lines. Cell lines are also characterized *via* transcriptome, genome-wide analysis of copy number gain/loss and sequences of 67 cancer-associated genes. Cancer Cell Line Encyclopedia [135] provides access to genomic, gene expression, chromosomal copy number and pharmacologic profiles of more than 1000 cell lines comprising 36 types of cancer. Cell-Miner resource [136] allows easy access to NCI-60 database compiled by the U.S. National Cancer Institute. This panel of 60 commonly used human cancer cell lines has been comprehensively characterized across various genomic, transcriptomic and pharmacologic platforms, including whole exome sequencing, several microarray platforms, and sensitivity to 20 000 compounds including 102 FDA-approved drugs.

We speculate that the broad employment of *in silico* analysis of cell lines data may be a promising option for personalizing drug treatment. It is cheaper and faster compared to performing *in vitro* experiments like mouse xenograft models and allows screening hundreds of drugs in parallel, at the same time taking into account specific molecular profiles. However, cancer cell lines have several drawbacks including the difficulties modeling tumor heterogeneity and microenvironment [137]. Also, recent studies raise important questions regarding the poor reproducibility of results [138] and uncertain consistency across different sources of pharmacological data [139, 140].

Another aspect that should be taken into account is the impact of epigenetic mechanisms upon tumorigenesis such as inactivation of tumor suppressors *via* promoter methylation or histone modifications [141]. Although epigenetic data is rarely available for cancer cell lines, it may still be important for the selection of therapy, such as in case of colorectal cancer [142]. An interesting resource is dbEM database which compiles information about gene essentiality, mutation, copy number variation and expression level of epigenetic proteins from thousands of tumors and cancer cell lines [143]. For in-depth discussion of the possible role of epigenetic abnormalities in cancers, please refer to [144, 145].

## SYSTEMS BIOLOGY AND DATA INTEGRATION

For the selection of optimal pharmacotherapy for an individual patient, it is necessary to understand precisely which molecular mechanisms drive the tumor progression. This problem can be addressed with a systems biology approach - how to interpret expression and mutation data and take them to a higher level of understanding. Here we will briefly describe basic applications of systems biology and data integration, while more details on this topic could be found in other publications [146–148].

## BIOLOGICAL PATHWAY RESOURCES

The basis for the systems biology analysis is the biological knowledge represented in the form of relations between various molecular entities: genes/proteins, complexes, small molecules and etc. In its simplest form, this information can be represented as a collection of genesets, i.e. groups of functionally related genes. The most commonly used genesets can be obtained either *via* Gene Ontology annotations [149] or *via* MSigDB signatures [150]. More sophisticated expert knowledge is represented in the form of signalling and metabolic pathways describing specific biochemical processes. The most commonly used public pathway resources include KEGG PATHWAY [151], BioCarta [152] and Reactome [153]. Several databases accumulate information from a number of other pathway resources: ConsensusPathDB [154], PathwayCommons [155]. Finally, biological knowledge can be represented not as a set of separate pathways (whose boundaries are set more or less arbitrarily), but rather as a global network containing tens of thousands of entities interconnected by various types of physical and genetic interactions. Examples of such resources are STRING [156] and BioGRID [157].

The drug action/metabolism pathways describing pharmacokinetics and pharmacodynamics of a drug with potential pharmacogenetic associations are of particular value for the study of personalized medicine. This category of pathways can be downloaded from PharmGKB [121] or The Small Molecule Pathway Database [158]. Integration of mutation calls with drug pathways may identify proteins that can be targeted by the earlier approved drugs.

It should be noted that relatively few public resources provide сancer-specific pathways. Pathway Interaction Database [159] contains publicly available collection of curated and peer-reviewed pathways implicated in cancer. However, this database has not been updated since 2012. The Molecular Signatures Database stores hundreds of gene signatures which are often dysregulated in cancer. However, a majority of these signatures were generated directly from transcriptomics experiments rather than created by experts and hence may be unreliable. Network of Cancer Genes [160], although it is not a pathway resource in the literal sense, reports information on interactions, functions and expression of approximately 2000 of known and candidate cancer genes and oncomiRs.

A particularly valuable cancer-specific database is ACSN, Atlas of Cancer Signalling Networks [161]. This resource aims to provide comprehensive maps of signalling and regulatory molecular processes that are frequently deregulated during cancerogenesis underlying the cancer hallmarks [162]. The key idea of ACSN is to consider cancerogenesis at several hierarchical levels: from bird-eye view maps of the 5 biological processes such as cell cycle or DNA repair through 52 detailed functional modules down to the seamless global network with thousands of molecular interactions. The whole cancer signalling network can be browsed with Google Maps interface allowing various zoom levels.

Several pathway resources provide a web interface allowing to overlay researcher's own data on available pathways. For example KEGGViewer [163] and Reactome [153] provide tools for coloring pathways according to expression data. ACSN resource allows users to overlay any expression, copy-number and mutation data on cancer maps, facilitating its biological interpretation.

Standalone pathway applications usually provide richer functionality and enable more sophisticated types of system-biological analyses using pathways and networks. Among the free standalone applications the most frequently used tool is Cytoscape [164]. Cytoscape offers rich opportunities for visualization of biological pathways and networks and integrates them with any data attributes, including gene expression and copy-number variations. Cytoscape supports many formats for data exchange and can be extended by more than 200 community-developed plugins, covering a variety of systems biology algorithms (a good intro can be found in [165]). Other applications for pathway visualization include PathVisio (integrated with WikiPathways database) [166] and GenMAPP [167].

Commercial pathway packages, such as Ingenuity^®^ Pathway Analysis [27], MetaCore™ [168] and Pathway Studio^®^ [169] come with comprehensive and carefully curated proprietary molecular databases and produce visually appealing networks. All these products include support for functional analysis of NGS data.

## FUNCTIONAL INTERPRETATION OF DATA

One of the priorities in selecting personalized anticancer pharmacotherapy is to understand which specific signalling pathways are perturbed in an individual patient. Over the last decade, considerable efforts were undertaken in this direction, stimulated by the successes of microarray technology. The most simple approach (although the most popular one), the so-called over-representation analysis, consists of identifying genes with significantly altered expression in tumors followed by finding predefined genesets/pathways where the observed fraction of altered genes differs from the expected value. Discovery of such overrepresented genesets allows researcher to interpret expression data for the individual patient in terms of pathways.

Many tools have been developed for performing overrepresentation analysis including DAVID [170] and WebGestalt [171]. Most methods are commonly based on hypergeometric distribution (Fisher's exact test) and differ very slightly from each other [146]. The drawback of this approach is the necessity to determine differentially expressed genes, which is not a trivial task for NGS data [172]. It is possible to apply the overrepresentation analysis for other types of variations in order to identify pathways enriched by mutated genes. However, in this case, the results will be noisy due to passenger mutations, which comprise the majority of somatic genetic variations.

A more advanced class of methods, the so-called functional class scoring, works directly with all genes measured in an experiment that are ranked, for example, by the strength of differential expression. If the rank distribution of genes within a specific geneset significantly differs from the background, then this geneset is somehow activated. This approach provides greater sensitivity in detecting small, though coordinated, expression changes of functionally related genes. The most commonly used algorithm implementing functional class scoring is the gene set enrichment analysis, GSEA [173]. There are many extensions and improvements of this classical algorithm including single-sample oriented analysis like GSVA [174] and ssGSEA [175].

A natural extension of the above methods is to utilize additional biological knowledge presented in the form of relations between entities in the pathway. Several algorithms have been developed implementing this idea. One popular method, SPIA, combines standard overrepresentation test with a measure of the actual perturbation on a given pathway taking into account relative gene locations [176]. Another method, DEAP, identifies those pathways where observed expression data is better “explained” by activatory/inhibitory relations between genes [177]. Pathway topology-based algorithms claim to have better specificity and more sensitivity compared to classical approaches.

However, when used for NGS data, most of the traditional approaches for functional interpretation are prone to potential biases and should be applied with care. Given that the genetic alterations occur evenly across the genome, long genes tend to harbor more mutations. Hence, the results of over-representation analysis for the list of mutated genes will be biased for pathways containing longer genes than other pathways [178]. A similar effect is present in the analysis of differential expression of RNASeq data: longer transcripts generate a greater number of reads and are more likely to be detected as differentially expressed compared with their short counterparts [179]. Several enrichment-based algorithms explicitly take into account this long-gene effect, including GOSeq [180], SeqGSEA [181], GSVA [174], and GOglm [182].

## DATA INTEGRATION

The data integration, as a union of the results obtained by various omics technologies, is of special importance when dealing with cancer data. Biology of cancer cells is extremely complex with alterations occurring on (epi)genomic, transcriptomic, proteomic and metabolomic levels. Hence, in order to improve statistical and interpretative power and obtain reliable view of an individual's tumor biology, it is necessary to sum up the maximum possible number of sources of information, each capturing a different aspect of cancerogenesis.

There is no single standard approach to data integration. While several algorithms have been developed such as GSAA [183], iCluster+ [184] and GSOA [185], most of them are designed to deal with a cohort of samples and require large training sets. One of the best known methods of data integration is PARADIGM [186, 187] which utilizes CNA and gene expression in order to infer patient-specific genetic activities. An example of a true single-sample approach using multiple sources of data is PHIAL [116] - an algorithm for annotation and ranking a patient's somatic alterations on the basis of their clinical and biological relevance. This approach takes into account SNV data, CNAs, and chromosomal rearrangement, as well as intrasample pathway structure: a mutation located within a gene connected to other gene with a known actionable alteration receives higher score.

Data integration can also be performed by visualizing results of various analyses on the same plot. There are several ways to depict multidimensional oncogenomics data such as matrix heatmaps, genomic coordinates, and networks (see [188] for a comprehensive review). These plots can be built using various standalone applications and websites such as GItools [189], IntOGen [190], cBioportal[92], Cytoscape [164]. A commonly used option is circos plots [191], where the genomic coordinates of all chromosomes are represented in a circular layout and where additional data tracks may include mutation pattern, CNAs, genomic rearrangements etc.

Methods for identification of activated pathways can also be considered as an approach to data integration since they allow aggregating various types of molecular events across several genes in the common feature space, simplifying data interpretation and gaining insight into the biological system [147]. For example, consider a specific pathway which has been predicted as activated according to transcriptomics analysis and also contains a mutated transcription factor (for example, MYC) “explaining” observed changes in expression. This finding directly points to potential causal mechanisms for tumor progression and can give clues to the needed therapy.

## CONCLUSIONS

Since introduction of the first predictive cancer biomarkers to the clinics, much progress has been made in the area of Precision Oncology. The number of genes associated with therapy choice has grown significantly, and corresponding variation detection has started to require NGS application. The «Exceptional responders» approach has been developed to find new predictive biomarkers. The patients with the best response to the tumor-specific therapy are studied thoroughly using the methods of NGS in order to find characteristic molecular features.

However, using standard clinical trials design, it may be difficult to confirm the clinical importance of these new genomic variations because of their low frequency. To overcome this issue, new “basket” type of clinical trials with patients stratified on the base of tumor molecular profile only is being developed.

As the cost of sequencing decreases, another approach becomes more popular. In this approach, the molecular profile of each patient is studied as completely as possible, taking into account the specific properties of the studied tissues. The systems biology analysis and integration of different kinds of NGS data play a critical role for detection of the most probable targets for the personalized therapy. In this article, we reviewed some examples of the corresponding case-studies, the general approach to this kind of the data interpretation, and the specific instruments that can be used. Despite the fact that the existing examples are quite promising, further development and verification of standards in NGS data processing for Precision Oncology is still necessary.

## ABBREVIATIONS

aCGH: Comparative Genomic Hybridization
ALL: acute lymphoblastic leukemia
AML: Acute myeloid leukemia
CISH: chromogenic In situ hybridization
CMI: Caris Molecular Intelligence platform
CNAs: copy number alterations
DPD: dihydropyrimidine dehydrogenase
CpG (sites): regions of DNA where a cytosine nucleotide occurs next to a guanine nucleotide separated by only one phosphate
FDA: Food and Drug Administration
FFPE: fresh-frozen paraffin embedded block
FISH: Fluorescence in situ hybridization
G6PD: Glucose-6-phosphate dehydrogenase
IHC: immunohistochemistry
InDels: insertions and deletions
LDH: lactate dehydrogenase
LOH: Loss of heterozygosity
MDS: myelodysplastic syndromes
MPN: Myeloproliferative neoplasms
mtDNA: mitochondrial DNA
NCI: National Cancer Institute
NGS: new generation sequencing
NIH: National Institutes of Health
NSCLC: Non-small-cell lung carcinoma
PSA: prostate-specific antigen
RNA-Seq: RNA sequencing
RT-PCR: reverse transcription polymerase chain reaction
SNVs: single nucleotide variations
TPMT: thiopurine methyltransferase
WES: whole exome sequencing
WGS: whole genome sequencing
